# Rb‐independent E2F3 promotes cell proliferation and alters expression of genes involved in metabolism and inflammation

**DOI:** 10.1002/2211-5463.12306

**Published:** 2017-09-12

**Authors:** Yang Liao, Wei Du

**Affiliations:** ^1^ Ben May Department for Cancer Research The University of Chicago IL USA

**Keywords:** E2F, E2F3^LQ^, inflammation, metabolism, retinoblastoma, retinoblastoma‐independent E2F

## Abstract

E2F transcription factors are key targets of the retinoblastoma (Rb) tumor suppressor. Despite extensive studies, the *in vivo* consequences of disrupting the interaction between Rb and an individual E2F are not clear. Here, we report an E2F mutation that interfered with binding to Rb family proteins without significantly affecting protein level or transactivation function. Characterization of mouse embryonic fibroblasts with this Rb‐independent E2F3^LQ^ mutation revealed that disrupting the Rb and E2F3 interaction increased cell proliferation, allowed cells to accumulate to higher density, and significantly altered expression of genes involved in metabolism, inflammation, immunity, and response to stress. These results suggest that the Rb‐independent E2F^LQ^ mutations might provide useful tools to investigate the *in vivo* consequences of disrupting the interactions between Rb and E2F.

**Abbreviations**
DMEM: Dulbecco's modified Eagle's medium
IP: immunoprecipitation
MEFs: mouse embryonic fibroblasts
Rb: retinoblastoma

The retinoblastoma (Rb) tumor suppressor is a member of the Rb family that is often inactivated in cancers. The functions of Rb are believed to be mediated by the binding of Rb to a large number of cellular targets, particularly the E2F family of transcription factors 1. The E2F proteins can be subdivided into three groups: activating, repressive, and Rb‐independent 2, 3. There are three activating E2Fs (E2F1–3) in mammalian systems but only one activating E2F (dE2F1) in *Drosophila*. Generally speaking, Rb regulates E2F target gene expression both by binding to E2F, particularly the activating E2F proteins, and by recruiting the chromatin‐modifying enzymes to regulate target gene expression 4. Genome‐wide studies have revealed diverse functions of E2F targets, including DNA replication and repair, cell cycle regulation, cell cycle checkpoint, cell death, differentiation 5, 6.

While the large numbers of E2F targets potentially explain the diverse functions of Rb in the cell, particularly those related to the cell cycle, there are conflicting results about the roles of free E2F proteins (E2F proteins that were not bound by the Rb proteins) and their contribution to Rb inactivation‐induced developmental and tumor phenotypes. On the one hand, removing the transcriptional activation function of dE2F1 has been shown to rescue the lethality of fly Rb (*rbf*) mutant, resulting in the development of adult flies with normal‐looking appendages 7. Similarly, inactivation of either E2F1 or E2F3 can partially suppress the developmental defects of Rb^−/−^ mice phenotypes and suppress the pituitary tumor incidence of Rb^+/−^ mice 8, 9, 10, 11. Furthermore, the Rb1^R654W/+^ mutation, which corresponds to the Rb^R661W^ mutation identified from low penetrance Rbs, reduced the ability to bind E2F and promoted pituitary tumor development similar to the Rb^+/−^ mutation 12, 13, 14. These results suggest that inhibition of E2F activity is the critical function of Rb in normal and cancer development in both fly and mammalian systems. On the other hand, loss of either E2F1 or E2F3 in mice can also promote the development of certain cancers, suggesting that the activating E2F proteins also have tumor suppressor function 9, 11. In addition, the Rb^∆G^ mutation, which disrupts the binding between Rb and the E2F transcription activation domain, does not cause spontaneous tumor development despite deregulated E2F target gene expression and accelerated G1/S transition after serum induction 15. As cells with Rb mutations will have multiple free activating E2F, loss of Rb and repressive E2F4 complex, increased expression of Rb family member p107, which can bind activating E2F proteins in the absence of Rb 15, 16, we still do not know the exact *in vivo* consequences of having individual free activating E2F despite extensive studies of the Rb and E2F proteins. One approach to study the role of free E2F *in vivo* is to use E2F mutations that specifically disrupt the interaction with the Rb family of proteins without other effects. However as the Rb binding domain of E2F overlaps with its transcription activation domain 17, such E2F mutants can be difficult to identify.

dE2F1^su89^ was identified as an allele of dE2F1 that suppressed phenotypes of RBF overexpression 18. Sequence analysis revealed that dE2F1^su89^ has a point mutation in the conserved Rb binding domain of dE2F1 that changes a conserved Leu to Gln. Because the Rb binding domain of E2F overlaps with the transcription activation domain, mutations in this domain often also impacted the ability of E2F to activate transcription. Interestingly, the dE2F1^su89^ mutation disrupts dE2F1's interaction with RBF without affecting the transcription activation function and causes deregulated E2F target expression, particularly in regions with high dE2F1 levels such as the morphogenetic furrow of developing eye disk 18. Therefore, dE2F1^su89^ lost its regulation by RBF, which we referred to as Rb family‐independent E2F. As dE2F1^su89^ contains a single point mutation of the conserved L786 in the Rb binding domain of dE2F1, it is likely that the same point mutation in the corresponding mammalian activating E2F proteins will also generate the mammalian Rb‐independent E2Fs, which potentially provide ideal tools to characterize the consequences of disrupting the interactions between an individual E2F and the Rb family of proteins without affecting the function of other Rb targets.

In this study, we introduced the LQ mutation into the Rb binding domain of mammalian activating E2F proteins and showed that the LQ mutation impairs the ability of E2F proteins to bind Rb binding without significantly affecting the protein levels or the ability to activate transcription. We further introduced the LQ mutation into mouse E2F3, generated the corresponding E2F3^LQ^ mouse embryonic fibroblasts (MEFs), and characterized the consequences of having the Rb family‐independent E2F3 at cellular level.

## Materials and methods

### Plasmid construction

To make p107 (aa379–1104) expression construct, p107 (aa379–1104) was amplified by the primers p107 forward: 5′‐GGCGGATCCGAAAGAAGCAGTCATTACTCC‐3′ and p107 reverse: 5′‐GGCGGATCCCCTTACCTTAGAAGGGCTGC‐3′. The PCR fragments were digested and cloned into pET15b vector (Novagen, Billerica, MA, USA).

To make E2F1 L415Q‐mutant construct, fragment N was amplified by primers CMV forward: 5′‐CCTACAGCTCCTGGGCAACG‐3′ and E2F1 L415Q‐mutant reverse: 5′‐GCCCTCCTCCTGGCCGAAGTGG‐3′. Fragment C was amplified by primers E2F1 L415Q forward: 5′‐CCACTTCGGCCAGGAGGAGGGC‐3′ and CMV reverse: 5′‐AAGTCAAGGCTTTTCTATGG‐3′. The whole mutant fragment was amplified using fragment N and C mixture as a template by the CMV primers. The final PCR product was digested by *Bam*HI and ligated into vector pCMV‐HA‐Bam. The wild‐type (WT) E2F1 was amplified by CMV primers and cloned into pCMV‐HA‐Bam vector.

For E2F2 L416Q construct, fragment N was amplified by primers E2F2 forward: 5′‐GACGGATCCATGCTGCAAGGGC‐3′ and E2F2 L416Q‐mutant reverse: 5′‐CACCCGCCTCCTGGCCCCACAGG‐3′. Fragment C was amplified by E2F2 L416Q‐mutant forward: 5′‐CCTGTGGGGCCAGGAGGCGGGTG‐3′ and pGEX‐2T *Bam*HI reverse: 5′‐GGCGGATCCCACCCAACTGATCTTCAGCATC‐3′. The whole mutant fragment was amplified using fragment N and C mixture as a template by the primers E2F2 forward and pGEX‐2T *Bam*HI reverse. The final PCR product was digested by *Bam*HI and cloned into pCMV‐HA‐Bam vector. The E2F2 was amplified by primers E2F2 forward and pGEX‐2T *Bam*HI reverse and cloned into pCMV‐HA‐Bam vector.

For E2F3 L438Q construct, fragment N was amplified by primers E2F3a CDS forward: 5′‐CGTGGATCCGCCACCATGGACAAAAGGGCACTG‐3′ and E2F3a L438Q‐mutant reverse: 5′‐CCTCCTCCCCCTGGCTCAGGAGATAG‐3′. Fragment C was amplified by E2F3a L438Q‐mutant forward: 5′‐CTATCTCCTGAGCCAGGGGGAGGAGG‐3′ and pGEX‐2T *Bam*HI reverse. The whole mutant fragment was amplified using fragment N and C mixture as a template by the primers E2F3a CDS forward and pGEX‐2T *Bam*HI reverse. The final PCR product was digested by *Bam*HI and cloned into pCMV‐HA‐Bam vector. The E2F3 was amplified by the primers E2F3a CDS forward and E2F3a CDS reverse: 5′‐CGTGGATCCTCAACTACACATGAAGTCTTCC‐3′. The PCR product was digested by *Bam*HI and cloned into pCMV‐HA‐Bam vector. All above constructs were verified by sequencing.

GST‐pRb (aa379–928) is from the previous study 19.

### Generation of *E2F3* ^*LQ/+*^ mice

To generate E2F3^LQ^‐knockin mice, C57B6/T mouse ES cells were electroporated with the E2F3 targeting vectors shown in Fig. 3A. ES cells were selected by G418 by Southern blotting. Southern blot probes were generated from PCR amplification. Primers used: 5′ probe forward: 5′‐AGACCAGCCCTTCTACATAATGAG‐3′, 5′ probe reverse: 5′‐TGGACTCTCTTCTTCAAATCTCAGG‐3′; 3′ probe forward: 5′‐TTCTGCTGCTCGAAGCTGTTG‐3′, 3′ probe reverse: 5′‐ACCTGACCGCATCCTGAGAAG‐3′. The ES clones with the desired E2F3 mutation were injected into blastocysts in the transgenic core facility to generate chimeras, which were back‐crossed to generate mice with germline transmission. Germline‐transmitted mice were bred with *Mox2Cre* mice 20, to remove the floxed neocassette and generate mice with only the targeted E2F3^LQ^ mutation. To further validate the mutations introduced in E2F3^LQ^ mice, we sequenced E2F3 mRNA from *E2F3* ^*LQ/LQ*^ MEFs derived from the mice, and only the desired point mutations were found. The offspring were genotyped by PCR. The following primers were used: E2F3 LQ forward: 5′‐GACGTCGACTGGCTGTGTCTTAACCAAATGC‐3′, E2F3 LQ reverse: 5′‐CGTGACGCGGCCGCAGGACACAGATCACCATTAGGC‐3′; Cre common primer: 5′‐GGGACCACCTTCTTTTGGCTTC‐3′, Cre WT primer: 5′‐AAGATGTGGAGAGTTCGGGGTAG‐3′, Cre‐mutant primer: 5′‐CCAGATCCTCCTCAGAAATCAGC‐3′. All animals were housed and treated in accordance with protocols approved by the Institutional Animal Care and Use Committee (IACUC) at the University of Chicago.

### Generation of *E2F3* ^*LQ/LQ*^ MEFs and cell culture

Mouse embryonic fibroblast cells were isolated from 13.5‐day‐old embryos from *E2F3* ^*LQ/+*^ mice intercross according to the standard procedures. MEF and HEK293 cells (ATCC) were cultured in Dulbecco's modified Eagle's medium (DMEM) supplemented with 10% heat‐inactivated FBS and 50 units·mL^−1^ penicillin/streptomycin.

### Protein purification

GST‐pRb was purified as described 21. Purified protein was dialyzed for 2 h at 4 °C in dialysis buffer (25 mm Tris pH 7.5, 50 mm NaCl, 1 mm EDTA, 1 mm DTT, 10% glycerol). The concentration was determined by the OD280 and the calculated protein extinction coefficients. The protein was aliquoted and kept at −80 °C.

HIS‐p107 construct was transformed into BL21 (DE3). The single colonies were inoculated and grown at 30 °C. Bacteria pellet was resuspended in lysis buffer (50 mm NaH_2_PO_4_, 300 mm NaCl, 10 mm imidazole, pH 8.0). After sonication, the supernatant was incubated with NI‐NTA beads for 2 h. The mixture was loaded into a disposable column. After washing with wash buffer (50 mm NaH_2_PO_4_, 300 mm NaCl, 20 mm imidazole, pH 8.0), HIS‐tagged proteins were eluted with elution buffer (50 mm NaH_2_PO_4_, 300 mm NaCl, 250 mm imidazole, pH 8.0). The eluted protein was then dialyzed and determined as GST‐pRb protein.

### Fluorescence polarization binding assay

The fluorescence polarization assay was carried out according to Ref. 22. Briefly, Tamra‐labeled E2F1 WT peptide (5‐TAMRA‐EALDYHFGLEEGEGIRDLFDCDFG; United BioSystems, Herndon, VA, USA) and E2F1‐mutant peptide (5‐TAMRA‐EALDYHFGQEEGEGIRDLFDCDFG; Peptide 2.0, Chantilly, VA, USA) were dissolved in assay buffer (20 mm HEPES, 100 mm KCl, 0.1% Tween‐20, 5 mm DTT, pH 7.8), diluted to 100 nm, and plated into black 384‐well plates (Thermo Fisher Scientific, Waltham, MA, USA) at 1 μL per well. Purified proteins were serially diluted 1 : 2 into buffer for 11 times. For the interaction assay, 9 μL of each protein concentrations was added to the peptide‐containing 384‐well plates resulting in a final volume of 10 μL. The samples were incubated for 20 min in the dark at room temperature, and fluorescence polarization was measured by Analyst GT multimode reader (Molecular Devices, Sunnyvale, CA, USA) with excitation and emission wavelengths of 530 nm and 590 nm, respectively. Experimental values were output as millipolarization units and imported into Excel file where Michaelis–Menten kinetics equation was used to determine dissociation constants (*K*_d_) for each protein/peptide pairing by least‐squares nonlinear regression.

### Transient transfection

Cells from the exponential growth phase were seeded into plates the day before transfection. The cells were transfected by calcium phosphate coprecipitation method or PolyJet (SignaGen Laboratories, Rockville, MD, USA). After the cells were exposed to the transfection mixture for 6 h, the medium was replaced with fresh medium. The cells were incubated for 48 h before the cells were harvested for assay.

### Transcriptional reporter assay

Cells were plated into 24‐well plate to reach 70% confluence. The cells were transfected with indicated transfection mixture. Cell extracts were prepared at 48 h post‐transfection, and the luciferase activity was measured using Dual Luciferase Reporter Assay System (Promega, Madison, WI, USA) according to the manufacturer's protocol. Luciferase activity was read on a BD Monolight 3010 Luminometer (BD Biosciences, San Jose, CA, USA). All data points presented are the average measurement of three independent transfections.

### Western blot and IP

For western blot, cell or tissue samples were lysed in RIPA buffer (50 mm Tris/HCl pH 8.0, 150 mm NaCl, 0.1% SDS, and 0.5% Na deoxycholate, 1% NP‐40) with fresh proteinase inhibitor. Western blot data were acquired using a Li‐Cor Odyssey image reader. The following antibodies were used: β‐actin (AC‐15, dilution 1 : 3000; Santa Cruz, Dallas, TX, USA), E2F3 (C‐18, dilution 1 : 1000; Santa Cruz), pRb (4.1, 1 : 10; Hybridoma bank, Iowa City, IA, USA), HA (12CA5, dilution 1 : 10), goat anti‐mouse IRDye (dilution 1 : 10 000; Li‐Cor, Lincoln, NE, USA).

For immunoprecipitation (IP) experiments, cells were lysed in NP40 buffer (50 mm Tris/Cl pH 8.0, 150 mm NaCl, 5 mm EDTA, 15 mm MgCl_2_, 1 mm DTT, 0.1 mm NaF, 1% NP‐40) with fresh protease inhibitors. The clear supernatant was incubated with primary antibody for 2 h at 4 °C. After incubation, prewashed Protein G beads (GE healthcare, Little Chalfont, UK) were added to the samples, and the mixture was incubated for additional 2 h at 4 °C. After the incubation, the beads were intensely washed. Then, the beads were mixed with loading buffer and boiled for western blot.

### BrdU labeling

Passage 4 MEF cells were plated on coverslips in six‐well plates. After 48 h, the cells were washed twice with 1× PBS and then incubated in DMEM containing 0.1% FBS for 72 h. Then, the cells were fed with DMEM containing 10% FBS. At each time point, the cells were incubated with medium containing 3 mg·mL^−1^ BrdU for 2 h at 37 °C. The cells were fixed and permeabilized as described 23. After denaturing DNA with 2 N HCl, the cells were incubated with mouse anti‐BrdU antibody (G3G4, dilution 1 : 20, Hybridoma Bank). FITC anti‐mouse secondary antibody (dilution 1 : 200, Jackson ImmunoResearch, West Grove, PA, USA) was used for detection.

### Cell cycle analysis

Cells were collected and fixed in cold 70% ethanol. The fixed cells were treated with RNase for 30 min and then stained with propidium iodide (PI) solution (Sigma, St. Louis, MO, USA) at room temperature. Approximately 10 000/sample cells were analyzed using LSRII 3–8 flow cytometer and facsdiva software (BD Biosciences, San Jose, CA, USA).

### RNA isolation and qRT‐PCR

Total RNA was isolated from cells or mouse tissue using TRIzol (Invitrogen, Carlsbad, CA, USA) according to the manufacturer's instructions. 2 μg of total RNA was reverse‐transcribed using M‐MLV reverse transcriptase (Promega) and random primers. PCR was performed in triplicate using SYBR green mix (Biotool, Houston, TX, USA) qPCR condition used: 3 min at 95 °C followed by 45 cycles of 95 °C for 20 s, 60 °C for 30 s, and 65 °C for 1 min. The primers are listed in Table S3.

### Microarray analysis

Cells were cultured and collected in the indicated condition. Total RNA was extracted with miRNeasy Mini Kit (Qiagen, Hilden, Germany). Microarray was carried out with Illumina Mouse WG‐6 array at the functional genomics facility at the University of Chicago. Illumina GenomeStudio module for gene expression analysis was used to analyze the microarray data.

The microarray data have been deposited to the GEO database. Accession number is GEO GSE102379.

### Statistics

*P* values were determined using the Student's unpaired *t*‐test.

## Result and Discussion

### An L‐to‐Q mutation in the conserved Rb binding domain impairs the binding between E2F1 peptide and the Rb family proteins

**Figure 1:**
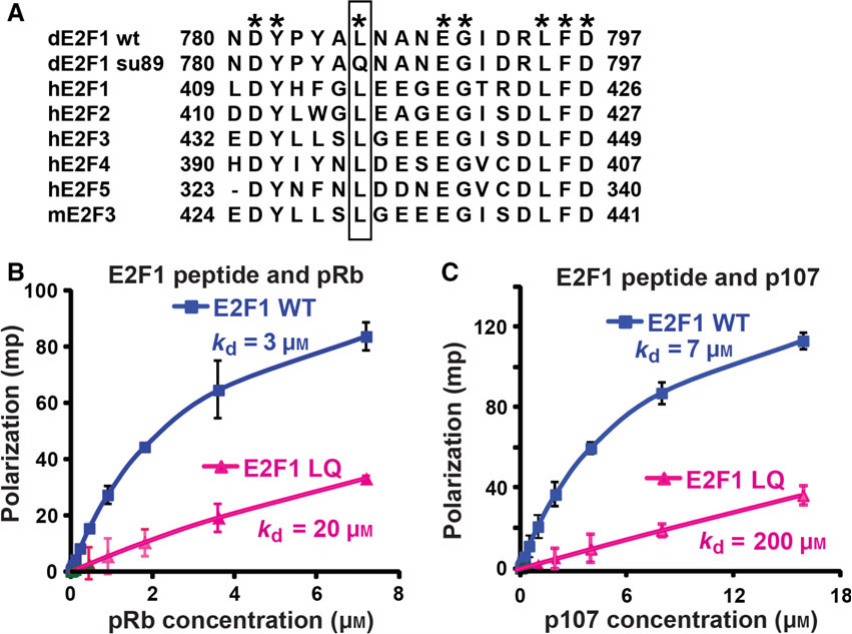
Mutation of the conserved L to Q in Rb binding domain of E2F1 significantly impaired the interaction with both Rb and the Rb family protein p107. (A) Sequence alignment of the Rb binding domain of E2F proteins. The conserved amino acids were labeled with star. The conserved leucine mutated in E2F^su89^ mutant is shown in rectangle. dE2F1 wt, wild‐type Drosophila E2F1; dE2F1 su89, Drosophila E2F^su89^ mutant; hE2Fs, human E2Fs; mE2F3, mouse E2F3. (B,C) Fluorescence polarization assays to determine the binding between TAMRA‐labeled E2F1 WT or L415Q peptide (LQ) and natively purified Rb family proteins pRb (B) or p107 (C). Experimental values were shown as millipolarization units (mp). Michaelis–Menten kinetics equation was used to determine the *K*_d_ for each protein/peptide pairing by least‐squares nonlinear regression.

Alignment of the Rb binding domain of E2F revealed that the Leu mutated in dE2F1^su89^ is highly conserved in all the activating and repressive E2F proteins (Fig. 1A) 18. Our previous report showed that the LQ mutation disrupted the interaction between fly dE2F1 and fly Rb without significantly affecting dE2F1 protein levels, the binding to dDP, and the ability to activate transcription 18. Indeed, dE2F1^su89^ can activate E2F target gene expression even in the presence of overexpressed fly Rb. Therefore, the LQ mutation in dE2F1^su89^ renders it Rb‐independent. We hypothesized that the same LQ mutation in the conserved mammalian activating E2F proteins may have similar effects.

To characterize the effects of the LQ mutation in mammalian E2F proteins, we first compared the effect of this LQ mutation on the binding between the E2F1 C‐terminal peptides and Rb family proteins using the fluorescence polarization binding assay 24. Synthesized TAMRA‐labeled WT or L415Q‐mutant E2F1 peptides were incubated with purified Rb family proteins pRb or p107 and the level of fluorescence polarization was determined. The *K*_d_ was calculated and used for comparing the binding activity. Consistent with the fact that E2F1 binds preferentially to Rb than to p107 25, the *K*_d_ between WT E2F1 peptide and pRb was around 3 μm, while the *K*_d_ between WT E2F1 peptide and p107 was 7 μm (Fig. 1B,C). Interestingly, the *K*_d_ between the LQ‐mutant peptide and Rb was 20 μm (Fig. 1B), indicating that the LQ mutation significantly interfered the binding between the E2F1 peptide and Rb protein *in vitro*. Furthermore, the *K*_d_ between the E2F1 LQ‐mutant peptide and p107 was 200 μm (Fig. 1C), indicating that the LQ mutation has even more dramatic effect on the binding of E2F1 to the Rb family protein p107. Therefore, the LQ mutation significantly interferes the interaction between the E2F1 peptide and Rb family proteins *in vitro*.

### The L‐to‐Q mutation interferes the binding between all the activating E2F proteins and Rb

**Figure 2:**
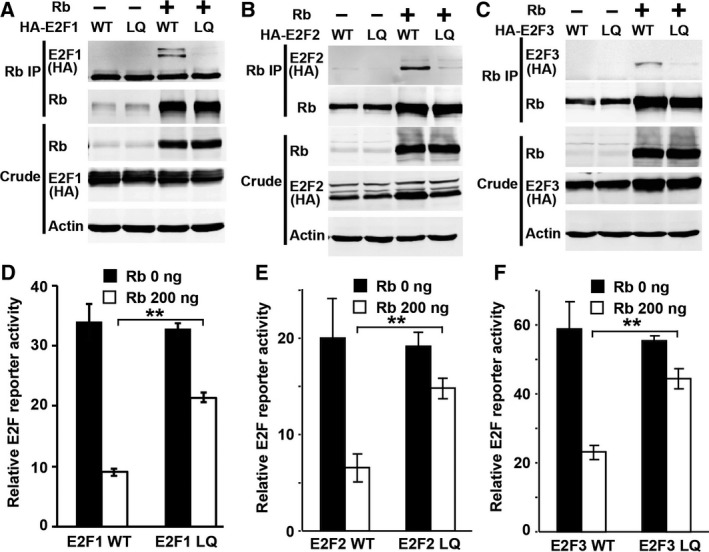
The LQ mutations in E2F1, E2F2, or E2F3 disrupt their interaction with Rb without affecting the transcription activation function. (A–C) IP/western blot assays to determine the binding between full‐length Rb and WT or LQ‐mutant E2F proteins. The HA‐tagged E2F1 (A), E2F2 (B), or E2F3 (C) was cotransfected with Rb into HEK293T cells. The transfected cells were collected for Rb IP and assayed by western blots using the anti‐HA antibody. (D–F) Luciferase assays of transcription activation by WT or LQ‐mutant E2F proteins with or without cotransfected Rb in HEK293 cells. The E2F and E2F4B‐luc reporter were cotransfected with or without Rb. The transfected cells were used for luciferase assay. The E2F luciferase reporter activity for E2F1 (D), E2F2 (E), and E2F3 (F) was shown. All graphs represent mean ± SD. **P < 0.01.

There are three activating E2F proteins, E2F1–3, in mammalian cells. To further characterize whether the same LQ mutation affects the binding of these activating E2F with Rb in the context of full‐length E2F proteins, we generated full‐length human E2F1^L415Q^‐, E2F2^L416Q^‐, and E2F3^L438Q^‐expressing constructs as well as the corresponding WT controls. HEK293T cells were cotransfected with Rb and WT or mutant E2F^LQ^ constructs and the binding between Rb and E2F was determined by IP and western blot experiments. As shown in Fig. 2A–C, while WT and LQ‐mutant forms of E2F were expressed at similar levels, WT but not the LQ‐mutant forms of E2F1, 2, and 3 were significantly brought down by Rb IP (Fig. 2A–C). Therefore, the LQ mutation interfered the binding between Rb and all the activating E2Fs in the full‐length context.

### The LQ mutation does not affect the ability of E2F proteins to activate transcription

The Rb binding domain of E2F overlaps with the transactivation domain. Mutations in E2F that interfere its binding to Rb often affect its ability to activate transcription. We further tested the effect of LQ mutations on the ability of E2F to activate transcription and on their regulation by Rb. WT or LQ‐mutant E2F were transfected into HEK293 cells with or without cotransfected Rb and the level of an E2F reporter activity was measured. In the absence of cotransfected Rb, WT and LQ‐mutant version of E2F1, E2F2, or E2F3 induced similar level of E2F reporter activation (Fig. 2D–F). In addition, while Rb significantly inhibited transactivation by WT E2F, the ability of Rb to inhibit transactivation by the E2F1^LQ^‐, E2F2^LQ^‐, or E2F3^LQ^‐mutant proteins was significantly impaired (Fig. 2D–F). Taken together, these results indicate that the LQ mutations in E2F1, E2F2, and E2F3 do not affect their transcription activation function but significantly impairs their binding to and regulation by the Rb family of proteins.

### Generation of the *E2F3* ^*LQ*^‐knockin MEFs

**Figure 3:**
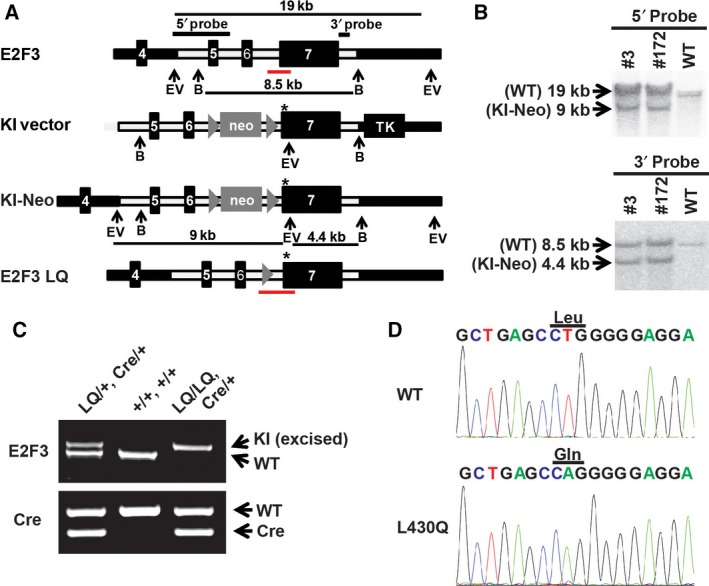
Generation of *E2F3* ^*LQ*^ mice and MEFs. (A) Targeting scheme for the generation of E2F3^LQ^ mice. The targeting construct contains a 5′ homology region with exons 5 and 6, a floxed neomycin‐resistant gene cassette (NEO), and a 3′ homology region with exon 7. * designates the LQ mutation. Red lines indicate PCR‐amplified regions in E2F3^WT^ and E2F3^LQ^‐knockin (KI) mice that are used for genotyping. Targeted ES clones were selected by G418 and detected by Southern blot with the indicated probes. Dark lines show the size of the fragments generated from digestion. B, *Bgl*II; EV,* Eco* RV. Mice generated from correctly targeted ES clones were mated with *Mox2Cre* mice to excise the NEO cassette and generate mice with the targeted E2F3^LQ^ mutation. (B) Southern blot analysis of targeted ES clones DNA digested by *Bgl*II and *Eco* RV and hybridized with the 5′ and 3′ probes shown in (A). For 5′ probe, correctly targeted clones #3 and #172 show a WT 19‐kb *Eco* RV band and a recombinant 9‐kb *Eco* RV band. For 3′ probe, correct clones #3 and #172 show a WT 8.5‐kb *Bgl* II band and a recombinant 4.4‐kb *Eco* RV and *Bgl* II bands. (C) PCR genotyping of the E2F3^LQ^‐mutant allele. The E2F3 WT and LQ‐mutant (KI) PCR products are 800 bp and 900 bp, respectively. The WT and Cre (Cre) PCR products are 410 and 300 bp, respectively. (D) Sequencing analysis of E2F3 transcripts expressed from WT and *E2F3* ^*LQ*^ (LQ) MEF cells. The L‐to‐Q mutation at L430 of E2F3 was labeled.

The above results suggest that the LQ mutation of E2F provides the perfect model to characterize the *in vivo* consequences of disrupting the Rb and activating E2F interaction. E2F3 is known to regulate cellular proliferation and previous studies showed that loss of E2F3 decreased proliferation in MEFs 26. Therefore, we generated an E2F3^LQ^‐knockin construct, which includes the LQ mutation, a nearby *Eco*RV site, and a neomycin cassette (Fig. 3A). After electroporation and G418 selection, correctly targeted *E2F3* ^*LQ*^ ES cell lines were identified by Southern blot (Fig. 3B). Two independent ES cell lines (#3 and #172) were used and generated two independent lines of E2F3^LQ^‐knockin mouse. The germline *E2F3* ^*LQ/+*^ mice were further crossed with *Mox2Cre* mice to excise the neomycin selection marker. The different genotypes were identified by PCR of genomic DNA (Fig. 3C). The LQ mutation in the E2F3 mice was also confirmed by sequencing, which clearly showed the targeted mutation of L (CTG) to Q (CAG; Fig. 3D). The E2F3^LQ/LQ^‐mutant mice are viable and can be obtained at Mendelian ratio. E2F3^LQ/LQ^ and WT littermate control embryos were obtained from *E2F3* ^*LQ/+*^ crosses at embryonic day 13.5 and were used to generate E2F3^LQ/LQ^ and WT control MEFs.

### *E2F3* ^*LQ/LQ*^ MEF cells showed increased expression of E2F target genes, early onset of DNA synthesis after serum stimulation

**Figure 4:**
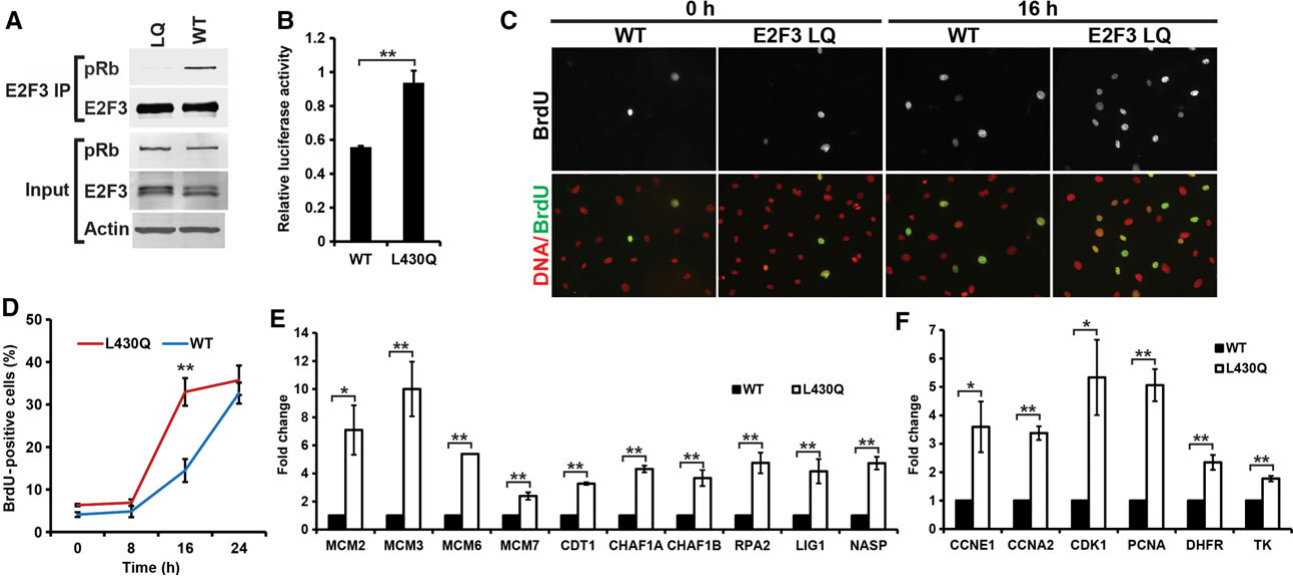
*E2F3* ^*LQ*^ ^*/*^ ^*LQ*^ MEF cells showed increased expression of cell cycle regulators and DNA replication factors in serum‐starved cells and promoted early onset of DNA synthesis after serum stimulation. (A) IP/western blot analysis of the interaction between endogenous E2F3 and pRb in WT and *E2F3* ^*LQ*^ (LQ) MEF cells. Whole cell lysates were processed for E2F3 IP. Immunoblot assay of Rb in E2F3 IP was shown. (B) Luciferase analysis of E2F transactivation activity in asynchronous WT and *E2F3* ^*LQ*^ MEF cells. The E2F4B‐luc was transfected into the MEF cells. The transfected cells were used for luciferase assay. (C) DNA synthesis analysis of WT and *E2F3* ^*LQ*^ MEF cells by BrdU assay. After serum starvation for 72 h, serum was readded. At 0, 8, 16, and 24 h, cells were pulsed with 10 μm BrdU for 2 h, and then, cells were fixed and processed for immunofluorescence analysis for BrdU (green). The nuclei were stained with DAPI (blue). The 0‐h and 16‐h images were shown. (D) Quantification of BrdU labeling of WT and *E2F3* ^*LQ*^ MEF cells in (C). (E) Quantitative RT‐PCR analysis of DNA replication factors expression (including *Mcm2*,* Mcm3*,* Mcm6*,* Mcm7*,* Cdt1*,* Chaf1a*,* Chaf1b*,* Rpa2*,* Lig1,* and *Nasp*) in serum‐starved WT and *E2F3* ^*LQ*^ MEF cells. (F) Quantitative RT‐PCR analysis of several additional E2F target genes expression (including *Ccne1*,* Ccna2*,* Cdk1*,* Pcna*,* Dhfr,* and *Tk*) in serum‐starved WT and *E2F3* ^*LQ*^ MEF cells. All the experiments were performed with the MEF cells before passage 5. All graphs represent mean ± SD. *P < 0.05; **P < 0.01.

Western blot results showed that E2F3^LQ^ mutation did not significantly affect the E2F3 protein levels (Fig. 4A). IP/western blot results showed that significantly decreased level of Rb was coimmunoprecipitated with anti‐E2F3 antibody in the *E2F3* ^*LQ/LQ*^ MEFs (Fig. 4A). These observations confirm that the E2F3^LQ^ mutation significantly impairs its interaction with Rb at endogenous protein levels. Consistent with this, transfection of an E2F reporter into the WT or *E2F3* ^*LQ*^ MEFs showed that *E2F3* ^*LQ*^ MEFs exhibited increased E2F activity (Fig. 4B). As the best known E2F target genes include DNA replication factors and cell cycle regulators, are generally repressed in G1, and induced at G1/S transition, we further examined the effect of E2F3^LQ^ mutation on expression of such genes in 72‐h serum‐starved WT or *E2F3* ^*LQ*^ MEFs. Indeed, the expressions of known E2F regulated genes involved in cell cycle control and DNA replication, such as *Cyclin E*,* Cyclin A2*,* Cdk1*,* Pcna*,* Dhfr*,* Tk*,* Mcm2/3/6/7*,* Cdt1*,* Chaf1a/b*,* Rpa2*,* Lig1,* and *Nasp,* were all significantly increased in the *E2F3* ^*LQ*^ MEFs compared to the WT control MEFs (Fig. 4E,F).

Previous studies showed that *Rb* ^*−/−*^ MEFs enter S phase earlier than WT control after serum stimulation 27 and the *E2F3* ^*−/−*^ MEFs showed delayed G1/S transition and reduced rate of DNA synthesis 26. Therefore, we further characterized the effect of E2F3^LQ^ mutation on the rate of S‐phase entry and cell proliferation. WT and *E2F3* ^*LQ*^ MEFs were serum‐starved for 72 h and then stimulated to enter the cell cycle by the addition of serum. BrdU incorporation was used to follow WT and *E2F3* ^*LQ*^ MEF cell cycle entry. Significant levels of BrdU incorporation was first detected at 16 h after serum addition in WT MEFs, which peaked at 24 h. In contrast, significantly higher levels of BrdU‐incorporating cells were observed in the *E2F3* ^*LQ*^ MEFs at 16 h (over 35% in *E2F3* ^*LQ*^ MEFs compared with 15% in WT MEFs; Fig. 4C,D). These data suggest that the Rb family‐independent E2F3 is sufficient to promote earlier onset of DNA synthesis after serum stimulation.

### *E2F3* ^*LQ/LQ*^ MEF cells proliferate faster, reach higher cell density, and have increased expression of genes involved in metabolism, inflammation, and response to stress

**Figure 5:**
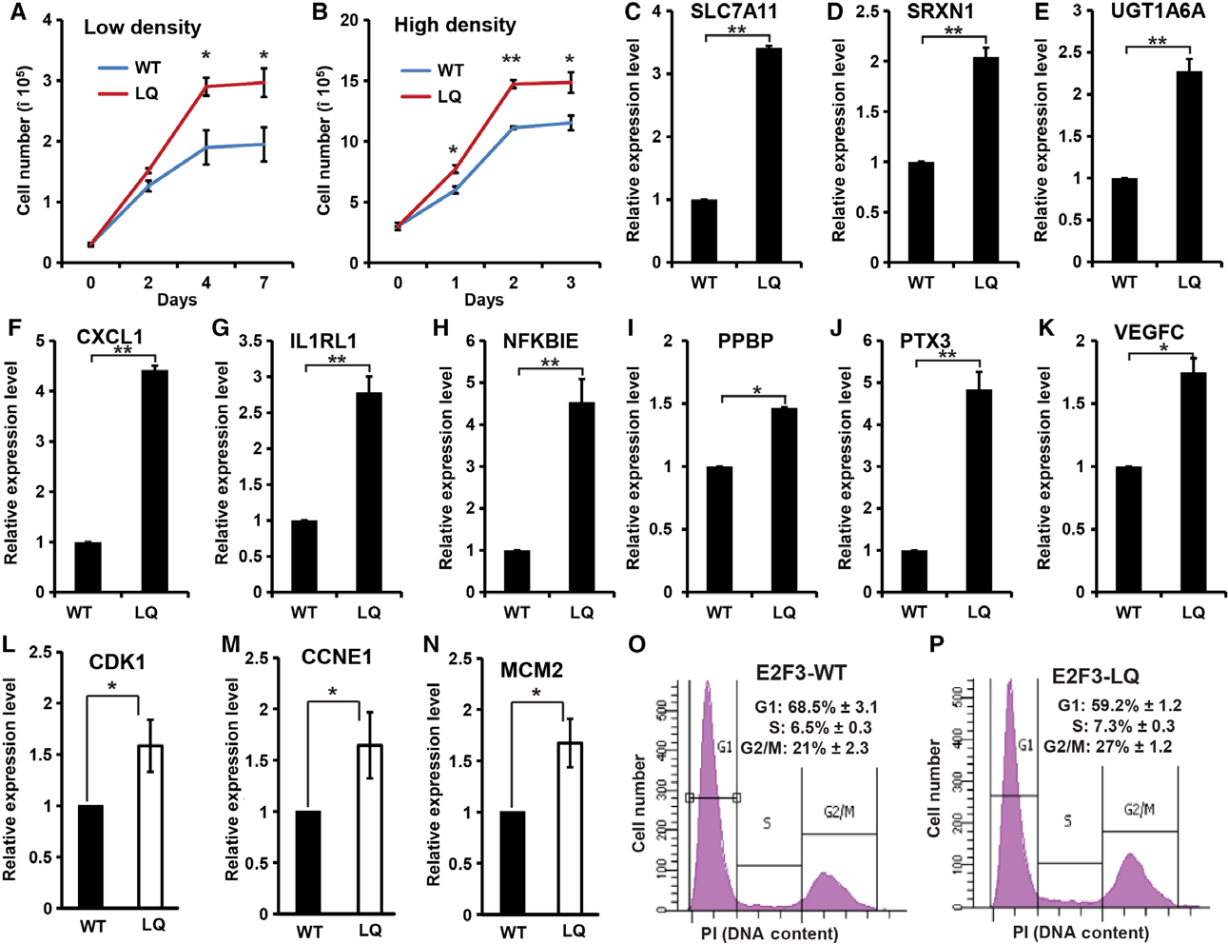
*E2F3* ^*LQ*^ ^*/*^ ^*LQ*^ MEF cells proliferate faster, reach higher cell density, and show increased expression of genes involved in metabolism, inflammation, and response to stress. (A,B) Cell growth rate analysis of WT and *E2F3* ^*LQ*^ MEF cells. Cells were seeded in the low density (0.3 × 10^5^) (A) or high density (3 × 10^5^) (B) at day 0 and cell number was determined daily. (C–K) Expression of several genes involved in metabolism and inflammation was analyzed by quantitative RT‐PCR in asynchronous proliferating WT and *E2F3* ^*LQ*^ MEF cells. (L–N) Expression of several cell cycle and DNA replication targets of E2F was analyzed by quantitative RT‐PCR in asynchronous proliferating WT and *E2F3* ^*LQ*^ MEF cells. (O–P), cell cycle profiles of WT (O) and *E2F3* ^*LQ*^ (P) MEFs were determined by FACS analysis. All the experiments were performed with the MEF cells before passage 5. All graphs represent mean ± SD. *P < 0.05; **P < 0.01.

In our attempt using 3T3 protocol to establish the spontaneously immortalized cells from WT and *E2F3* ^*LQ*^ MEFs, we noticed that the *E2F3* ^*LQ*^ MEFs appear to proliferate faster and accumulated more cells. Cell cycle analysis of the WT and *E2F3* ^*LQ*^ MEFs showed that the *E2F3* ^*LQ*^ MEFs had reduced proportion of G1 cells and increased proportion of S/G2 cells (Fig. 5O–P). In addition, determination of the cell proliferation rate confirmed that the *E2F3* ^*LQ*^ MEFs exhibit significantly increased rate of cell proliferation and reached higher cell density than that of WT MEFs when cultured under either high‐ or low‐density conditions (Fig. 5A,B). The increased cell proliferation of the *E2F3* ^*LQ*^ MEFs also made it easier to establish the *E2F3* ^*LQ*^ cell lines based on 3T3 protocol.

The higher rate of cell proliferation and increased cell density suggest that *E2F3* ^*LQ*^ affects other functions in addition to cell cycle and DNA replication. Microarray experiments were carried out using RNA from asynchronously proliferating WT and *E2F3* ^*LQ*^ MEFs. A total of 134 genes were found to show significantly increased expression in *E2F3* ^*LQ*^ MEFs in comparison with the WT control MEFs (Table S1). As expected, one of the main functions affected by *E2F3* ^*LQ*^ mutation is related to metabolism, which includes multiple components of the transport proteins, ROS and redox regulators, and metabolic enzymes (Table S2). Unexpectedly, the top functions affected by *E2F3* ^*LQ*^ mutation are related to inflammation, immunity, and response to stress (Table S2). These include multiple cytokines, chemokines, NF‐КB regulators, modulators and targets of proinflammatory signaling, and angiogenesis regulators. The expression of some of the genes involved in these categories was further characterized by RT‐PCR. As shown in Fig. 5C–K, expressions of SLC7A11 (a component of anionic amino acid transport system that is highly specific for cysteine and glutamate), SRXN1 (sulfiredoxin, a redox protein), UGT1A6A (UDP glucuronosyltransferase), CXCL1 (chemokine), IL1RL1 (a member of the interleukin 1 receptor family), NF‐КBIE (inhibitor of NF‐КB), PPBP (CXC family chemokine), PTX3 (pentraxin 3, which is induced by inflammatory cytokines and serves as a biomarker for several inflammatory conditions), and VEGFC (VEGF, angiogenesis) are all significantly increased in the proliferating *E2F3* ^*LQ*^ MEFs. It should be pointed out that the *E2F3* ^*LQ*^ MEFs still had higher expression of the cell cycle and DNA replication targets of E2F, such as CCNE1, CDK1, and MDM2, in the asynchronously proliferating MEFs (Fig. 5L–N), although the difference in expression is much less than those observed in the serum‐starved MEFs observed in Fig. 4E–F. This is not surprising as these E2F targets are repressed in G1 but are highly expressed in S/G2 cells in WT MEFs.

Although several E2F1 mutants were reported previously that disrupt the interactions with Rb, one report showed that the identified E2F1 mutant expressed higher protein levels and the ability to activate E2F target gene was reduced in C‐33A cells 19 and the other report showed that most of the mutants moderately reduced reporter activation, although only one CAT assay was shown and the protein levels were not determined 28. In this report, we tested the hypothesis that the conserved E2F^LQ^ mutation that makes E2F^su89^ to be Rb‐independent in flies 18 will also make the mammalian activating E2Fs to be Rb‐independent. The results shown in this report strongly support this idea. Therefore, the E2F^LQ^ mutations will be nice tool to investigate the *in vivo* consequences of disrupting the interactions between Rb family and an individual E2F.

The observed increased cell cycle regulator and DNA replication factor expression in *E2F3* ^*LQ*^ MEFs are consistent with the more rapid G1/S transition of the *E2F3* ^*LQ*^ MEF cells. Interestingly, *E2F3* ^*LQ*^ MEFs increased rate of MEF proliferation and accumulated at high cell density, suggesting that E2F3^LQ^ affects cell growth and cell communication in addition to S phase. Characterizing the expression of genes altered in *E2F3* ^*LQ*^ MEFs revealed that genes involved in metabolism, inflammation, immunity, and response to stress were significantly affected. It is interesting to note that cell proliferation in both the WT and *E2F3* ^*LQ*^ MEFs leveled off before reaching confluence when MEFs were seeded in low cell density (Fig. 5A–B). We speculate that the cessation of MEF proliferation under such low‐density culture condition is not due to contact inhibition but rather due to the limiting amount of factors, including factors secreted by MEFs. An interesting possibility is that the secreted factors regulating inflammation and stress response enriched in *E2F3* ^*LQ*^ MEFs may contribute to the observed increased proliferation of *E2F3* ^*LQ*^ MEFs. Further studies will be needed to determine how E2F3^LQ^ leads to the activation of stress and inflammation‐related genes and whether this affects normal and cancer development.

## Author contributions

YL performed the experiments, analyzed data and prepared the figures. WD designed and supervised the experiments, and edited figures and wrote the manuscript.

## Supporting information


**Table S1**. List of genes that have increased expression in E2F3LQ MEFs.
**Table S2**. GO analysis of genes that have increased expression in E2F3LQ MEFs.
**Table S3**. List of primers used for qRT‐PCR.
