# Supplementary material for: Rb‐independent E2F3 promotes cell proliferation and alters expression of genes involved in metabolism and inflammation
Source: FEBS Open Bio. 2017 Sep 12;7(10):1611–21. doi: 10.1002/2211-5463.12306 (PMC5623693; doi:10.1002/2211-5463.12306)
Supplement: Supplementary file 1 — Table S1. List of genes that have increased expression in E2F3LQ MEFs. Table S2. GO analysis of genes that have increased expression in E2F3LQ MEFs. Table S3. List of primers used for qRT‐PCR. [file FEB4-7-1611-s001.pdf]

Supporting Information

Table S1. List of genes that have increased expression in *E2F3*^{LQ} MEFs.

**Table S1.** List of genes that have increased expression in *E2F3*^{LQ} MEFs.					
A4GALT	CX3CL1	GVIN1	MAP3K8	RGS16	TMEM176A
AA467197	CXCL1	H2-T23	MAPK13	RGS3	TMEM176B
ADAR	CXCL16	HMOX1	MGST1	RIPK2	TNIP1
ADARB1	CYP7B1	HP	MICALL2	SAMD9L	TOR3A
ADSSL1	D14ERTD668E	IER3	MLKL	SERPINA3G	TREX1
ALDOC	DCN	IFI35	MMP13	SERPINA3H	TRIM21
ANGPTL4	DCXR	IFIT2	MMP3	SERPING1	UBE1L
ANGPTL7	DGAT2	IFIT3	MOV10	SLC11A2	UBE2L6
AQP1	DHX58	IGTP	MT2	SLC25A37	UGT1A10
BC004044	DPEP1	IIGP2	NFKBIA	SLC2A6	UGT1A6A
BC006779	EG277333	IKBKE	NFKBIE	SLC7A11	USP18
BCL3	EGLN1	IL1RL1	NFKBIZ	SLC7A2	VEGFA
BST2	ENPP4	LGALS9	NQO1	SLCO3A1	VEGFC
CASP4	ESD	LOC100038882	OASL2	SLCO4A1	ZC3H12A
CBR2	ESM1	LOC100044190	OSBPL3	SNCG	
CCDC85B	FAM162A	LOC100044204	PARP12	SOD2	
CCL2	GBP2	LOC100046120	PARP14	SOD3	
CCL4	GBP3	LOC100047963	PDK1	SRXN1	
CCL7	GCH1	LOC100048346	PPBP	STAT1	
CD47	GDF15	LOC667370	PPFIA3	STAT2	
CD55	GLRX	LRIG1	PRDX5	TAP1	
CH25H	GREM1	LUM	PTGES	TAPBP	
CISH	GSDMDC1	LY6E	PTX3	TCIRG1	
CP	GSTM1	MACROD1	RARRES2	TLR2	

Table S2. GO analysis of genes that have increased expression in *E2F3^LQ* MEFs.

Table S2. GO analysis of genes that have increased expression in *E2F3^LQ* MEFs.

GO Term	P-Value	Genes
GO:0006950 Response to stress	7.84E-21	Pdk1 Hmox1 Cx3cl1 Trex1 Hp Tapbp Cxcl1 Nfkbiz Slc11a2 Fam162a Vegfa Tnip1 Adar Rarres2 Prdx5 Irgm2 Mapk13 Aqp1 Ier3 Gch1 Gbp2 Egln1 Angptl4 Nqo1 Mgst1 Ifit3 Oasl2 Ifit2Enpp4 Gbp3 Cxcl16 Slc7a2 Ccl2 Cd55 Cd47 Stat1 Tlr2 Serping1 Tap1 Srxn1 Bcl3 Macrod1 Ikbke Nfkbia Mt2 Bst2 Ripk2 Sod2 Il1rl1 Sod3 Ccl4 Slc7a11 Ccl7 Dhx58
GO:0002376 Immune system process	6.64E-16	Hmox1 Cx3cl1 Ptx3 Tapbp Cxcl1 Slc11a2 Vegfa Tnip1 Adar Rarres2 Irgm2 Vegfc Gch1 Gbp2 Ifit3 Oasl2 Ifit2 Gbp3 Cxcl16 Tmem176a Grem1 Slc7a2 Ccl2 Cd55 Cd47 Stat1 Tlr2Serping1 Tap1 Bcl3 Nfkbia Bst2 Ripk2 Sod2 Il1rl1 H2-T23 Ccl4 Ccl7 Dhx58 Tmem176b
GO:0006955 Immune response	5.23E-15	Hmox1 Cx3cl1 Cxcl1 Vegfa Tnip1 Adar Irgm2 Gch1 Gbp2 Ifit3 Oasl2 Ifit2 Gbp3 Cxcl16 Ccl2 Cd55 Stat1 Tlr2 Serping1 Tap1 Bcl3 Nfkbia Bst2 Ripk2 Il1rl1 H2-T23 Ccl4 Ccl7 Dhx58
GO:0045087 Innate immune response	3.64E-13	Tnip1 Adar Irgm2 Gch1 Gbp2 Ifit3 Oasl2 Ifit2 Gbp3 Cxcl16 Ccl2 Cd55 Stat1 Tlr2 Serping1 Tap1 Nfkbia Bst2 Ripk2 Dhx58
GO:0008152 Metabolic process	1.02E-11	Dcn Casp4 Adarb1 Pdk1 Hmox1 Cp Dgat2 Usp18 Trex1 Nfkbie Mlkl Ptx3 A4galt Hp Ugt1a10 Map3k8 Tapbp Gstm1 Nfkbiz Slc11a2 Fam162a Vegfa Tnip1 Adar Rarres2 Prdx5 Mmp3Dpep1 Irgm2 Mapk13 Vegfc Zc3h12a Aqp1 Ugt1a6a Ier3 Gch1 Gbp2 Egln1 Angptl4 Tor3a Nqo1 Mgst1 Rgs3 Mov10 Parp14 Stat2 Oasl2 Enpp4 Gbp3 Lum Cyp7b1 Uba7 Grem1 Slco4a1Slc7a2 Ptges Aldoc Ccl2 Cd55 Rgs16 Stat1 Tlr2 Trim21 Serping1 Tap1 Glrx Srxn1 Bcl3 Macrod1 Ikbke Cbr2 Nfkbia Esd Ch25h Helz2 Bst2 Phf11d Ly6e Serpina3g Ripk2 Ube2l6 Mmp13Sod2 Adssl1 Igtp Dcxr Sod3 Ccl4 Ccdc85b
GO:0034097 Response to cytokine stimulus	2.04E-08	Cx3cl1 Trex1 Adar Irgm2 Gch1 Gbp2 Ifit3 Ifit2 Gbp3 Cxcl16 Ccl2 Stat1 Bst2 Serpina3g Ripk2 Il1rl1 Igtp
GO:0044237 Cellular metabolic process	1.53E-07	Dcn Adarb1 Pdk1 Hmox1 Dgat2 Usp18 Trex1 Nfkbie Mlkl Ptx3 Hp Map3k8 Tapbp Gstm1 Nfkbiz Slc11a2 Vegfa Tnip1 Adar Rarres2 Prdx5 Dpep1 Irgm2 Mapk13 Vegfc Zc3h12a Aqp1Ugt1a6a Ier3 Gch1 Gbp2 Egln1 Tor3a Nqo1 Mgst1 Rgs3 Mov10 Parp14 Stat2 Oasl2 Gbp3 Lum Cyp7b1 Uba7 Grem1 Slco4a1 Slc7a2 Ptges Aldoc Rgs16 Stat1 Tlr2 Trim21 Tap1 Bcl3 Macrod1 Ikbke Cbr2 Nfkbia Esd Ch25h Helz2 Phf11d Ly6e Ripk2 Ube2l6 Mmp13 Sod2 Adssl1 Igtp Dcxr Sod3 Ccl4 Ccdc85b
GO:0035456 Response to interferon-beta	1.88E-07	Trex1 Gbp2 Ifit3 Gbp3 Stat1 Bst2 Igtp
GO:0007249 I-kappaB kinase/NF-kappaB cascade	2.94E-07	Hmox1 Lgals9 Tnip1 Grem1 Stat1 Tlr2 Bcl3 Ikbke Nfkbia Bst2 Ripk2 Il1rl1
GO:0009611 Response to wounding	3.06E-07	Hmox1 Cx3cl1 Hp Cxcl1 Nfkbiz Tnip1 Aqp1 Ier3 Enpp4 Slc7a2 Ccl2 Cd55 Cd47 Tlr2 Serping1 Sod2 Il1rl1 Ccl4 Slc7a11 Ccl7
GO:0034341 Response to interferon-gamma	6.88E-07	Irgm2 Gch1 Gbp2 Gbp3 Cxcl16 Ccl2 Stat1 Bst2
GO:0030595 Leukocyte chemotaxis	1.22E-06	Cx3cl1 Cxcl1 Vegfa Rarres2 Vegfc Cxcl16 Grem1 Ccl2 Ccl4 Ccl7
GO:0006979 Response to oxidative stress	4.41E-06	Pdk1 Hmox1 Hp Slc11a2 Prdx5 Aqp1 Egln1 Nqo1 Mgst1 Srxn1 Sod2 Sod3
GO:0002687 Positive regulation of leukocyte migration	8.22E-06	Cx3cl1 Vegfa Rarres2 Vegfc Ccl2 Tlr2 Ccl4 Ccl7
GO:0006954 Inflammatory response	9.76E-06	Hmox1 Hp Cxcl1 Nfkbiz Tnip1 Ier3 Slc7a2 Ccl2 Cd55 Cd47 Tlr2 Serping1 Il1rl1 Ccl4 Ccl7
GO:0043122 Regulation of I-kappaB kinase/NF-kappaB cascade	2.16E-05	Hmox1 Lgals9 Tnip1 Grem1 Stat1 Tlr2 Nfkbia Bst2 Ripk2 Il1rl1
GO:1901342 Regulation of vasculature development	9.74E-04	Hmox1 Cx3cl1 Vegfa Vegfc Aqp1 Egln1 Grem1 Ccl2 Stat1

Table S3. List of primers used for qRT-PCR.

**Table S3.** List of primers used for qRT-PCR		
**Gene Name**	**Forward (5'-3')**	**Reverse (5'-3')**
CCNE1	GGAAGACTCCCACAACATCC	GTCTCCTGCTCGCTGCTCTG
CCNA2	GCTTCAGCTTGTAGGCACGG	ACTGTTGGTGCAGCCAAGTC
CDK1	GTCCGTCGTAACCTGTTGAG	AGTCTGATCTTCTTCATGGC
PCNA	CTGCAAGTGGAGAGCTTGGC	GTAGGAGACAGTGGAGTGGC
DHFR	AGAACCACCACGAGGAGCTC	CACAAAGAGTCTGAGGTGGC
TK1	CCTGACATTGTGGATTTCTGTG	TTCTCGGAAGCACTCCATGC
MCM2	AGATCCACCACCGCTTCAAG	AGGAAGTATGCCAACACGTGC
MCM3	TGGCTTTCCCATCCAGTTCTG	TCTGGCATCTCCTGGATGGTG
MCM6	AGATCTGTCATACAGGCTGGTC	TCTTAATGCTCTCAGCGGTCTG
MCM7	ACATCACCTATGTCCACCAGC	TGTGGGCTGTCTCTCGTGGC
NASP	AGGACAGAGAAAATGACAAGGC	AGCATATCCCAGGCAAGTTCC
CHAF1A	TCCAGGAGGGAAAAGAGGGACC	TGCGGCATTGCTCCTGGAATTC
CHAF1B	TGGAAGATCCACAGGCTGGC	TGGTGTGTCGAGCAAGATTGG
CDT1	AACCTGGTGGAACGTGTCAAGG	ATGTCAGGCACCTCGTCCAC
LIG1	AGTGCCAGCTGACCCACTCAC	GGAACACAGTCTCTACCATTGC
RPA2	TCCCAGCTGCTTTCTGCTACTC	GAGCCTTCTCCGCATGTCTG
IL1RL1	TTCTGGATTGAGGTTGCTCTG	GGAAGACCCGAGGGTAAATG
NFKBIE	GCCTCAACAGCATCTCATCC	TCGAAGGGCAAATAAGAAAGG
PPBP	CTCAGACCTACATCGTCCTGC	AGCGCAACAAGGATCAGGC
PTX3	CCTGCGATCCTGCTTTGTG	GGTGGGATGAAGTCCATTGTC
SLC7A11	ATCTTCGATACAAACGCCCA	AACCATGAAGAGGCAGGTGA
SRXN1	CCCAGGGTGGCGACTACTA	GTGGACCTCACGAGCTTGG
UGT1A6A	GTTTCTCTTCCTAGTGCTTTGGG	CCTCGTTCACTGAGATGTTCTAC
VEGFC	TGCAGTGCATGAACACCAG	TGATTGTGACTGGTTTGGGG
CXCL1	TGCCTTGACCCTGAAGCTC	AGACAGGTGCCATCAGAGCAG
GAPDH	GCACAGTCAAGGCCGAGAAT	GCCTTCTCCA TGGTGGTGAA
